# Electrical detection of the flat-band dispersion in van der Waals field-effect structures

**DOI:** 10.1038/s41565-023-01489-x

**Published:** 2023-08-17

**Authors:** Gabriele Pasquale, Edoardo Lopriore, Zhe Sun, Kristiāns Čerņevičs, Fedele Tagarelli, Kenji Watanabe, Takashi Taniguchi, Oleg V. Yazyev, Andras Kis

**Affiliations:** 1 Institute of Electrical and Microengineering, École Polytechnique Fédérale de Lausanne (EPFL), Lausanne, Switzerland; 2 Institute of Materials Science and Engineering, École Polytechnique Fédérale de Lausanne (EPFL), Lausanne, Switzerland; 3 Institute of Physics, École Polytechnique Fédérale de Lausanne (EPFL), Lausanne, Switzerland; 4 Research Center for Functional Materials, National Institute for Materials Science, Tsukuba, Japan; 5 International Center for Materials Nanoarchitectonics, National Institute for Materials Science, Tsukuba, Japan

**Keywords:** Nanoscale devices, Two-dimensional materials

## Abstract

Two-dimensional flat-band systems have recently attracted considerable interest due to the rich physics unveiled by emergent phenomena and correlated electronic states at van Hove singularities. However, the difficulties in electrically detecting the flat-band position in field-effect structures are slowing down the investigation of their properties. In this work, we use indium selenide (InSe) as a flat-band system due to a van Hove singularity at the valence-band edge in a few-layer form of the material without the requirement of a twist angle. We investigate tunnelling photocurrents in gated few-layer InSe structures and relate them to ambipolar transport and photoluminescence measurements. We observe an appearance of a sharp change in tunnelling mechanisms due to the presence of the van Hove singularity at the flat band. We further corroborate our findings by studying tunnelling currents as a reliable probe for the flat-band position up to room temperature. Our results create an alternative approach to studying flat-band systems in heterostructures of two-dimensional materials.

## Abstract (web summary)

The energy position of the van Hove singularity in a van der Waals flat-band system is detected by tunnelling (photo)currents within a field-effect structure.

## Main

Flat-band systems have recently become an exciting playground for studying strongly correlated phenomena and emergent physics in two-dimensional (2D) materials. After the discovery of correlated insulating and superconducting states in twisted bilayer graphene^1^, the quest for materials systems exhibiting exotic phenomena has quickly spread to twisted bilayer TMDCs^2,3^. Another material that has a flat band in its pristine form is indium selenide (InSe)^4^, a layered 2D semiconductor belonging to the III–VI metal monochalcogenide family. It possesses distinctive electrical^5,6^ and optical properties^7,8^, making it an attractive platform for optoelectronic devices based on 2D materials^9–12^.

The bandgap of γ-InSe changes from a direct bandgap of roughly 1.27 eV for bulk to a roughly 2.8 eV indirect bandgap in the monolayer limit^5,13,14^. The indirect-to-direct bandgap transition is due to a shift of the valence-band maximum (VBM) from the Γ point towards the K points. This band structure modification is accompanied by an appearance of a flat-band dispersion and a van Hove singularity in the density of states (DOS), and has been predicted to possibly induce ferromagnetism or superconductivity^15–17^. The possibility to study emergent phenomena in devices based on InSe is appealing because of a simpler device fabrication, which does not require twist-angle engineering, unlike in other platforms based on 2D materials^1–3^. While several reports have confirmed flat-band dispersions in 2D systems, only a handful of technically demanding methods such as ARPES and STM^4,18^ have so far been used to experimentally verify the presence of a singularity in the DOS. In particular, in the case of InSe, the flat valence band has not yet been detected using electrical transport measurements, and its presence has only been revealed by STS^7^ and ARPES^4^. The difficulty of detecting the flat band through electrical transport measurements is a hurdle for studying emergent physics in 2D materials.

Here, we use measurements of out-of-plane tunnelling currents in hexagonal boron nitride (hBN)-encapsulated few-layer InSe samples to detect the Fermi level reaching the valence-band edge via electrical measurements. The van Hove singularity provides a sudden availability of carriers that induces an abrupt change in the tunnelling processes across the hBN insulating barrier. We provide evidence for the electrical detection of the flat-band position in InSe by comparing tunnelling currents with lateral transport and photoluminescence measurements. In our devices, we achieve p-type conduction for different thicknesses of InSe (three and six layers, 3L and 6L) and show that the onset of the out-of-plane tunnelling current is consistent with the beginning of hole transport at cryogenic temperatures. We observe that lateral hole transport deteriorates for a decreasing number of InSe layers due to the high hole effective mass at the valence band^5^. This confirms lateral transport to be an ineffective way to probe flat-band physics when going towards few-layer systems with large valence-band dispersions. Instead, the magnitude of out-of-plane currents depends on the hole effective mass in the dielectric medium and not the semiconductor, while being directly related to the divergent DOS. This allows us to decouple the high availability of states from the large effective mass that hinders lateral transport, making tunnelling current measurements a practical approach for detecting the van Hove singularity. Furthermore, we directly relate different regimes in the gate-dependent tunnelling photocurrents to the behaviour of excitonic species in the photoluminescence spectra. In particular, we focus on the bandgap renormalization (BGR) effects appearing in the presence of a Fermi sea when the Fermi level is located inside the valence band^6^. The chosen range of InSe thicknesses (three to six layers) allows us to directly relate the tunnelling mechanisms to the lateral hole transport and photoluminescence in the flat-band regime^4^.

On the basis of our experimental evidence and theoretical models, we outline a complete picture that allows us to investigate a flat-band system and the accompanying evolution of the DOS as a function of thickness. Our work opens up the exploration of 2D flat-band systems and their emergent properties by using tunnelling photocurrents through dielectric barriers.

## Results

### Device structure and band diagrams of few-layer InSe

**Fig. 1:**
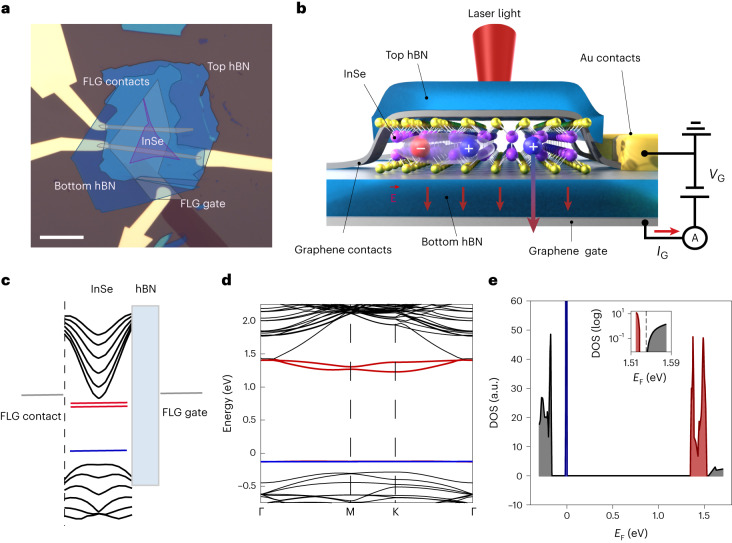
Schematic and band structure of an encapsulated InSe device. **a**, Optical microscope picture of the 3L device with layers highlighted in false colour. FLG, InSe and hBN layers are depicted in grey, violet and blue, respectively. Scale bar, 20 μm. **b**, Three-dimensional schematic representing an hBN-encapsulated InSe layer with graphene contacts and a wide back gate. The sample is optically excited using a red laser and excitonic species are formed within the semiconductor. When a gate voltage is applied, holes in InSe can tunnel through the bottom hBN giving rise to a tunnelling photocurrent. The electrical measurement scheme is shown on the right. **c**, Band alignment of the materials of interest in the device of Fig. 1b. Two electron donor (red) and one acceptor (blue) states are induced by the presence of selenium vacancies. **d**, Band structure of the 3L slab of InSe with a selenium vacancy. A van Hove singularity arises due to the band flattening at the Γ point. **e**, The DOS in a 3L-InSe shows a sharp peak at the VBM, as well as the donor (red) and acceptor (blue) states. The inset shows in logarithmic scale the region between the highest donor state and the beginning of the conduction band, with its minimum located at 1.54 eV with respect to the acceptor state.

To obtain high-quality samples, we encapsulate γ-phase InSe flakes in hBN and use few-layer graphite (FLG) flakes as electrodes. We use a FLG bottom gate to modulate the carrier density in the semiconductor. The complete device schematic is reported in Fig. 1b, representing an optically excited heterostructure based on InSe. In Fig. 1a, an example of a 3L InSe device is shown in false colour to highlight the stacked layers. The band structure for three layers of InSe was obtained by density functional theory (DFT) calculations accounting for the presence of selenium vacancies (*V*_Se_), the most common type of defects in few-layer InSe due to their low formation energy^19,20^ (Supplementary Note 1). These defects produce two peaks in the DOS close to the conduction band and a peak at roughly 150 meV from the valence band, as shown in Fig. 1c,d. In few-layer InSe, a sharp DOS increase characterizes the VBM position (Fig. 1e) due to the appearance of a van Hove singularity.

By taking defect states into account, we explain the nature of lateral hole transport in InSe devices and the change in tunnelling behaviour at the point corresponding to the flat-band position. Moreover, our calculations reveal valence-band offsets on the order of hundreds of meVs in heterostructures with FLG-contacted few-layer InSe on top of hBN (Fig. 1c and Supplementary Fig. 1), unveiling a notably asymmetrical alignment when compared to the band offsets at the conduction band (roughly 3.8 eV). This asymmetry is crucial for the electrical detection of the DOS at the van Hove singularity at the VBM.

We note that band alignments obtained by DFT calculations are known to be susceptible to variabilities^21^. Here, we use hybrid and meta-GGA functionals in DFT calculations to gain semiquantitative insights into the highly-asymmetrical band alignment between few-layer InSe and hBN. The calculated values of the band alignment are in the range of hundreds of meV for holes, which further confirms our findings regarding the order of magnitude of the barriers extracted experimentally from tunnelling photocurrent measurements, as presented in the following sections.

### Lateral transport and tunnelling photocurrent

**Fig. 2:**
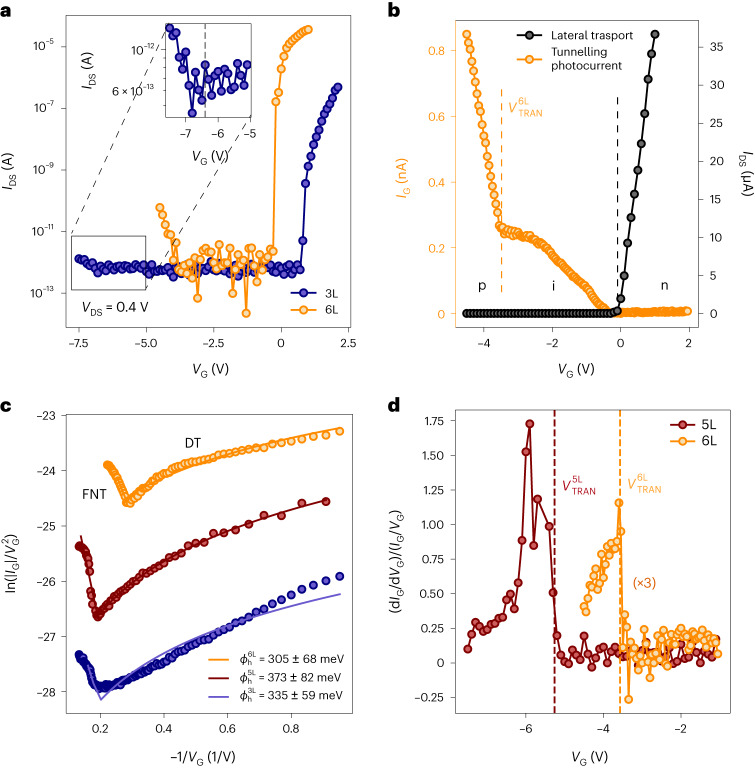
Field-effect transport and tunnelling photocurrent with few-layer InSe. **a**, Field-effect lateral transport in the 3L (measured at 80 mK) and 6L devices in logarithmic scale. Subthreshold p-type conduction was achieved in both samples. Note a lower subthreshold slope in the 3L with respect to the 6L device (Supplementary Note 2). The inset highlights the onset of p-type conduction in the 3L device (${V}_{\mathrm{ON}}^{3{\mathrm{L}}}\simeq-6.4\,{\mathrm{V}}$). **b**, The gate-dependent lateral current between the FLG contacts in a 6L device (black) shows predominantly n-type conduction in linear scale. The dashed grey line indicates the onset of n-type conduction. A gate-dependent tunnelling photocurrent is induced when the sample is illuminated using a 633 nm laser at 50 µW of power (yellow). The behaviour of the tunnelling current can be divided into two main regions, which are separated by a yellow dashed line with respect to the gate voltage. **c**, The gate-dependent tunnelling photocurrent data for 3L, 5L and 6L devices are reported in blue, red and yellow circles, respectively, using the $\mathrm{ln}\left(|{I}_{\mathrm{G}}\text{|}/{V}_{\mathrm{G}}^{2}\right)$ scale with respect to −1/*V*_G_ for *V*_G_ < −1 V. The tunnelling behaviour is modelled using Simmons’ approximation, with direct tunneling (DT) and FNT regimes separated by a sharp onset. FNT fittings reveal effective barriers for tunnelling holes of 237, 340 and 292 meV for 3L, 5L and 6L samples, respectively. **d**, Ratio between the differential conductance and the tunnelling conductance at low temperature for 5L and 6L devices. The weaker 3L signal does not allow us to observe changes in the normalized conductance at cryogenic temperature. Further discussion on the temperature dependence of the d*I*_G_/d*V*_G_ signals is provided in Supplementary Note 3.

On Fig. 2a we present the gate dependence of the lateral current in our 3L and 6L devices. We observe ambipolar transport with dominant n-type transport^5,6^ while p-type conduction is strongly suppressed due to relatively high hole effective masses compared to the electron ones (Supplementary Table 1). The successful observation of subthreshold p-type lateral currents in our devices is attributed to the combination of using a high-purity InSe crystal source (HQ Graphene) and full hBN-encapsulation with lateral graphene electrodes. This ensures that the InSe flakes are never exposed to air at any point during device handling (Methods). A visible decrease in the p-type subthreshold slope is seen in the 3L with respect to the 6L device, consistent with the reduction of the hole effective mass with an increasing number of InSe layers, as discussed in Supplementary Notes 1 and 2.

Illumination of the device with a laser beam (*λ* = 633 nm) results in the appearance of an out-of-plane tunnelling current between the InSe layers and the FLG gate, shown on Fig. 2b for the 6L device together with the lateral current as a function of gate voltage. The tunnelling current shows a rapid increase at *V*_G_ = −3.5 V, closely matching the hole conduction onset voltage *V*_ON_ ^6L^= −3.6 V. Photocurrents for devices with varying thicknesses (3L, five layers (5L) and 6L) are shown in Fig. 2c on a logarithmic scale, revealing two main regions with linear and logarithmic trends, respectively. This behaviour is reminiscent of the separation between direct tunnelling (DT) and Fowler–Nordheim tunnelling (FNT) mechanisms in electronic devices such as metal-insulator-metal diodes^22,23^ and can be modelled using Simmons’ approximation^22,24^ (Supplementary Note 3). To perform the fitting under Simmons’ approximation, we have used the measured bottom hBN thicknesses of 20, 25 and 18 nm for the 3L, 5L and 6L devices, respectively (Methods). The FNT part allows us to extract the tunnel barrier heights. We refer to the position of changing trends as the transition voltage *V*_TRAN_, which for the 6L device occurs at −3.5 V while for the 3L sample, ${V}_{\mathrm{TRAN}}^{3{\mathrm{L}}}\,$= $-5.8\,{\mathrm{V}}$, close to the onset of p-type conductivity ${V}_{\mathrm{ON}}^{3{\mathrm{L}}}\simeq-6.4\,{\mathrm{V}}$.

To rule out the possibility that the drastic change in the tunnelling transition is due to change in the transmission probability, we consider the differential tunnelling conductance d*I*_G_/d*V*_G_ normalized over the total conductance *I*_G_/*V*_G_ (Fig. 2d). As previously reported^25^, the appearance of a peak in the (d*I*_G_/d*V*_G_)/(*I*_G_/*V*_G_) curve indicates that the rise of the tunnelling signal is due to a sudden change in the material DOS (Supplementary Note 3).

### Relationship between tunnelling mechanisms and excitons

**Fig. 3:**
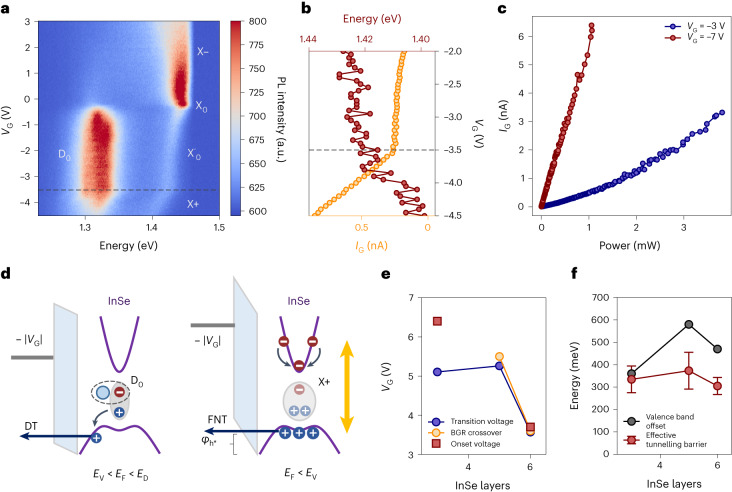
Excitonic emission and tunnelling mechanisms with few-layer InSe. **a**, Gate-dependent photoluminescence (PL) spectra for the 6L device. The change in the main excitonic peaks with respect to gate voltage is related to different charge configurations (Supplementary Note 4). When the Fermi level in the semiconductor enters the valence band (grey dashed line), the highest energy peak shifts linearly with respect to the gate voltage due to BGR in the presence of a dense hole Fermi sea. **b**, Peak position of the highest energy excitonic species (red) and tunnelling photocurrent (yellow) in the vicinity of the VBM. The direct tunnelling (DT)-to-FNT transition (${V}_{\mathrm{TRAN}}^{5{\mathrm{L}}}$) and the BGR start (${V}_{\mathrm{BGR}}^{5{\mathrm{L}}}$ are highlighted by dashed yellow and red lines, respectively. **c**, Power-dependent tunnelling photocurrent for the 5L device (Supplementary Note 5) when the Fermi level lies within the bandgap (blue) or has entered the valence band (red). The superlinear behaviour of the former case indicates that second-order excitonic effects play a role, which is excluded within the valence band. **d**, Auger and exciton–exciton recombination mechanisms can radiatively transfer their energy to resident carriers and induce hot-hole tunnelling through a thick hBN barrier in the presence of a vertical electric field (*E*_V_ *<* *E*_F_ *<* *E*_D_). When the Fermi level lies within the valence band (*E*_F_ *<* *E*_V_), excitonic species are subject to interactions with the Fermi sea induced by the high DOS and directly photo-excited holes are responsible for the photocurrent by FNT through the hBN barrier (right). **e**, Transition voltage (*V*_TRAN_), BGR crossover (*V*_BGR_) and p-type onset (*V*_ON_) for the 3L, 5L and 6L devices. The 5L sample was not equipped with two FLG contacts for lateral transport measurements, and the intensity of photoluminescence emission in the 3L sample was too low to be analysed as a function of gate voltage. **f**, Layer-dependent effective tunnelling barrier extracted from FNT fittings (Fig. 2b) and valence-band offsets obtained from DFT calculations (Supplementary Fig. 1). The effective barrier values are presented as mean values (dots) and corresponding standard deviations (error bars).

To understand the sharp transition in the tunnelling photocurrent in the presence of a van Hove singularity at the VBM, we compare tunnelling photocurrent measurements with gate-dependent photoluminescence spectroscopy (Fig. 3a), allowing us to identify different excitonic species and defect-bound states^8^. We focus our attention on the redshift of the main excitonic species in the p-type region X_*+*_, together with its strong suppression of emission for ${V}_{\mathrm{G}}^{6{\mathrm{L}}} < -3.5\,{\mathrm{V}}$. This corresponds to the Fermi level crossing into the valence band and the accompanying BGR at the flat band. This voltage also coincides with the onset of the tunnelling photocurrent in the 6L device (Fig. 3b) and hole accumulation in the semiconductor. Similar behaviour is shown in Supplementary Note 5 for the 5L device.

In Fig. 3c, we show the excitation power (*P*) dependence of the tunnelling photocurrent (*I*) in the p-type region (*V*_G_ = −7 V) and within the bandgap (*V*_G_ *=* *−*3 V), displaying a linear and a superlinear behaviour, respectively. *I* ∝ *P*^*α*^ dependence with *ɑ* ≅ 2 is commonly attributed to second-order mechanisms such as exciton–exciton annihilation^26^. For 1 < *α* < 2, first-order recombination pathways also play a role, mainly represented by Auger processes^27^. Here, non-radiative exciton recombination results in energy transfer to charge carriers, which become sufficiently excited to tunnel through the hBN barrier in the presence of a vertical electric field. The effective tunnelling barrier extracted for the Fowler–Nordheim region lies in the range of several hundred meV for 3L, 5L and 6L devices, with values that are comparable with the calculated valence-band offsets for the respective structures (Supplementary Fig. 1).

Previous reports have shown the excitation of charge carriers through Auger recombination and vertical photocurrent due to hot-carrier tunnelling for other 2D materials^24,27,28^. However, no DOS-dependent sharp transition between direct tunnelling and FNT regions can be identified in either our TMDC-based samples (Supplementary Note 6) or in the literature, where the increase in photocurrent is attributed to the bending of the hBN bands by an increasing electric field. A different mechanism is therefore needed to explain the sharp transition between direct tunnelling and FNT regions in InSe. In our case, the aforementioned suppression of photoluminescence from X_+_, together with the reduction of the power coefficient *α* with the onset of hole conduction, both indicate that exciton densities are reduced when the Fermi level enters the valence band. Since the generation factor is fixed only by the input optical power (Supplementary Note 4), we infer that, in the p-type region, Auger recombination plays a minor role in the tunnelling pathways from InSe through the hBN barrier.

Even if hot-carrier excitonic-assisted tunnelling were allowed in our system, it would not be a likely origin of the sharp onset in photocurrent since the effective barrier seen by holes is comparable to the one predicted by DFT (Fig. 3f). Directly photo-excited carriers have also been previously reported as a contribution to out-of-plane currents^26,29^. However, in our case, the magnitude of the extracted effective barrier indicates that tunnelling carriers are probed in the vicinity of the VBM and not from energy levels deep within the valence band (Fig. 3d). On the basis of our understanding of the power-dependent photocurrent and the BGR, we attribute the sharp onset of the photo-assisted process to a change of the main tunnelling species from hot carriers assisted by recombining defect-bound excitons (*V*_TRAN_ *<* *V*_G_ *<* 0 V) to directly photo-excited holes located at the VBM (*V*_G_ *<* *V*_TRAN_).

We note that the successful detection of the flat-band position by tunnelling mechanisms relies on the favourable band alignment between the van Hove singularity position and the dielectric barrier, as well as the thickness and out-of-plane carrier effective mass of the dielectric itself (Supplementary Note 8). In particular, we want to stress that further studies on the band alignment between dielectric materials and 2D flat-band systems are needed to apply tunnelling mechanisms for investigating their properties. Hence, the method proposed in this study can be successfully generalized to other systems, provided that the devices are engineered to obtain a sizeable exponential tunnelling current following the optimization of the aforementioned parameters.

### Room-temperature tunnelling current

**Fig. 4:**
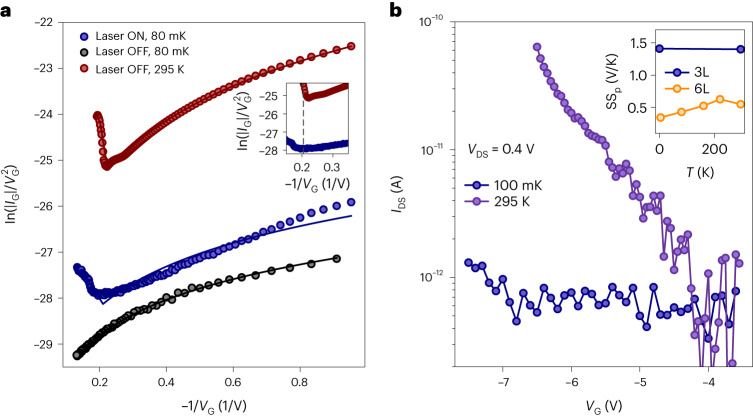
Temperature-dependent lateral transport and tunnelling current. **a**, Tunnelling current in the 3L device without laser illumination at 100 mK (black) and 295 K (red). The logarithmic dependence in the $\mathrm{ln}\left(|{I}_{\mathrm{G}}\text{|}/{V}_{\mathrm{G}}^{2}\right)$ scale with respect to −1/*V*_G_ for *V*_G_ < −1 V reveals no transition in the dark at low temperatures and a sharp transition at room temperature. In the inset, the tunnelling photocurrent at 100 mK (blue) is compared with the dark tunnelling current at room temperature, revealing a comparable trend in the Fowler–Nordheim regime (1/*V*_G_ < −0.2 V and a transition voltage of around 5 V for both cases. **b**, Lateral p-type transport for the 3L device at 100 mK (blue) and 295 K (violet). The thermal broadening due to Fermi–Dirac scaling with temperature induces a shift in the first-detectable subthreshold p-type signal^33^, as further discussed in Supplementary Note 2. However, no substantial change in the subthreshold slope is detected. In the inset, the p-type subthreshold slopes (SSp) of the 3L (blue) and 6L (yellow) devices are shown. The sizeable difference in magnitude of ${\mathrm{SS}}_{\mathrm{p}}^{3{\mathrm{L}}}$ and ${\mathrm{SS}}_{\mathrm{p}}^{6{\mathrm{L}}}$ both at room and cryogenic temperatures is related to the increase in hole effective mass in InSe for a decreasing number of layers.

The low exciton binding energy in InSe (roughly 10 meV)^12^, together with the desire to investigate emergent physics at the flat band^1,2^, motivated initial measurements performed at low temperatures. Extending the temperature range can further enhance our understanding of the tunnelling mechanisms in few-layer InSe. In Fig. 4a we show the tunnelling current measured without laser illumination at 80 mK (black) and 295 K (red). At 80 mK, the dark current exhibits a linear relationship with respect to *V*_G_, with no observable change in tunnelling mechanisms in the gate voltage range of interest. However, at room temperature we observe a sharp transition in tunnelling mechanisms from direct tunnelling to FNT. In fact, higher temperatures are related to an increase in tunnelling probabilities and in photocurrent signal magnitudes^30^. Moreover, the room-temperature transition voltage is consistent with that obtained at low temperatures under laser illumination (${V}_{\mathrm{TRAN}}^{\,\mathrm{RT}}\simeq 5\,{\mathrm{V}}$), as highlighted in the inset of Fig. 4a. This indicates that hole tunnelling from the VBM is dominating both at room temperature in the dark, where thermally enhanced out-of-plane transport of holes at the van Hove singularity is directly detectable, and at low temperatures with light, where directly photo-excited carriers near the VBM are extracted thanks to high electric fields. The temperature change only affects the magnitude of the detectable signal without influencing the origin of the tunnelling current. This confirms that the change in tunnelling mechanisms of out-of-plane currents is due to the Fermi level reaching the van Hove singularity in the DOS at the InSe VBM, and rules out excitonic effects.

We further observe a change in the lateral transport of holes at low and high temperatures (Fig. 4b). In fact, the presence of in-gap defect states influences the transport characteristics, as discussed in Supplementary Note 2. Due to the high hole effective mass (Supplementary Note 1), it is difficult to identify the valence-band edge from the onset of hole conduction in thinner InSe samples (*n* ≤ 3L). This explains the higher discrepancy in the quantities of interest shown in Fig. 3e between 3L and thicker samples. We can conclude that, while the onset of p-type conduction in our InSe devices is affected by the presence of defect states at room temperature, the tunnelling current transition voltage remains unaffected. Such a result supports the use of tunnelling currents as a reliable mechanism to electrically detect van Hove singularities of flat-band systems in field-effect devices.

## Conclusions

The precise detection of the flat band in field-effect structures at low temperatures is critical for exploring emergent phenomena such as magnetism or superconductivity^16,17^ and correlated electron states such as Wigner crystals and Mott insulators^2^. In this work, we have studied tunnelling currents in few-layer InSe and their relation to the van Hove singularity at the valence-band edge of the semiconductor (Supplementary Note 3). Although tunnelling photocurrents in van der Waals heterostructures have been previously observed, their exploration has been limited to photodetector applications and to studies of excitonic features in TMDCs^26–28^.

Since the presence of a flat dispersion gives rise to high effective carrier masses, the identification of lateral field-effect hole transport in InSe has been difficult, hindering the identification of the valence-band edge position in devices with a low number of layers. On the other hand, the magnitude of tunnelling currents is related to the effective mass of the holes in the insulating layer (hBN, 0.5*m*_0_)^24^, which is substantially smaller than that in InSe (0.931*m*_0_ for 6L). High signal-to-noise ratios in tunnelling photocurrents can be successfully achieved even in thin InSe (3L in Fig. 2c) by simply increasing the illumination intensity, resulting in increased carrier tunnelling. For example, while we see typical lateral currents in the 10 pA range (0.4 V in a 6L device at 4.5 K), we can observe tunnelling photocurrents of roughly 100 nA under similar conditions (Fig. 3c). While no observable damage to the structure is present with tunnelling photocurrents, the increase of lateral transport in the p-type region by gating is limited by dielectric breakdown^31^. Moreover, although the p-type onset changes with temperature due to defect states within the bandgap, the onset of the tunnelling current remains unchanged, providing a reliable marker for the flat-band position.

We have shown that the change in tunnelling mechanisms is related to the excitonic properties of the semiconductor, motivating the combined exploration of out-of-plane photo-assisted tunnelling with spectroscopy techniques in materials possessing a van Hove singularity. One notable advantage of exploiting photo-tunnelling in flat-band materials is that only two electrodes are needed to identify the position of the van Hove singularity at low temperatures, in our case the InSe contact and the bottom gate. Moreover, the method presented in this work could be applied to other flat-band systems to enrich the understanding of their emerging properties. We note that the successful use of tunnelling mechanisms with flat-band systems is dependent on careful device engineering, regarding in particular the choice of the tunnelling dielectric medium and its band alignment with the 2D materials of interest (Supplementary Note 8).

In light of our findings, few-layer InSe represents a reliable and highly reproducible platform to study flat-band physics in field-effect structures based on 2D materials^32^. Our results are expected to motivate the further exploration of flat-band materials and their properties by out-of-plane tunnelling currents, enriching the investigation of strongly correlated phenomena, correlated insulating states and emergent physics in van der Waals heterostructures.

## Methods

### Device fabrication

The devices used throughout this study are fabricated by the dry transfer technique. The FLG (NGS) and the bottom hBN are mechanically cleaved on silicon oxide. The bottom hBN flake is picked up with a polycarbonate membrane on polydimethylsiloxane and deposited on the FLG bottom gate. The few-layer InSe (HQ Graphene) flakes are further exfoliated on polydimethylsiloxane (Gelpak) to maximize the optical contrast. They are identified by optical contrast, and then transferred on the prepared hBN–FLG heterostack. Further, the top hBN and FLG electrodes are picked up in sequence, aligned and deposited on the InSe, thus encapsulating the air sensitive material. The whole process is carried out in an argon-filled glovebox (Inert) to avoid degradation or contamination. When capped, the heterostructure is annealed at 340 °C in a high vacuum at 10^−6^ mbar for 6 h. As a last step, the metal electrodes are made by e-beam lithography and metal evaporation (2/100 nm Ti/Au). The bottom hBN thicknesses are measured using atomic-force microscopy and used in our modelling as described in Supplementary Note 3. Further information on the fabrication and morphology of the devices can be found in Supplementary Note 7.

### Optical and electrical measurements

The measurements shown throughout this work were performed under vacuum at helium temperature unless stated otherwise. Photoluminescence measurements were carried out by shining a laser source onto the sample, focused on a spot with a diameter of about 1 µm. A narrow-linewidth tuneable continuous wave laser (MSquared) is used both for photoluminescence measurements and for tunnelling photocurrent measurements. The incident power was varied from 1 µW to 5 mW for power dependence measurements (Fig. 3c) and kept at 50 µW for the photoluminescence spectroscopy shown in Fig. 3a and tunnelling photocurrent measurements unless otherwise specified. Transport measurements were carried out at room temperature, helium temperature (4.2 K) and 80 mK with a Keithley 2636 sourcemeter. The 80 mK temperature was achieved inside a dilution fridge from Oxford Instruments, with a custom-made window and mirrors that allowed us to perform optical and optoelectronic measurements.

### First-principles calculations

Our first-principles calculations were performed using the VASP code^34^ at the DFT level. For all structure relaxations, semilocal Perdew–Burke–Ernzerhof functional^35^ was used. The conjugate gradient method was used to optimize atomic positions and lattice constants, where the total energy and atomic forces were minimized. The convergence was reached when the maximum force acting on each atom was less than 0.01 eV/Å, whereas the next energy step was smaller than 10^−5^ eV. For accurate bandgap estimations of pristine systems, the hybrid HSE06 (ref. ^36^) was used, whereas for defect calculations with large supercells, we used a modified Becke–Johnson exchange potential in combination with LDA correlation^37,38^. Kohn–Sham wave functions were expanded in a plane-wave basis set with a kinetic energy cut-off of 400 eV, while electron–core interactions were described through the projector augmented wave method^39,40^. All structures were subjected to periodic boundary conditions with a vacuum layer of 10 Å in the direction perpendicular to the layers to prevent interaction between replica images. The reported effective masses are obtained as an average between the two high-symmetry directions in the Brillouin zone, from Γ to K, and from Γ to M. We used a parabolic fitting, with a sampling of four points in the *k*-space to obtain the result (for further information see Supplementary Note 1). Defect calculations were carried out using 4 × 4 supercells. Finally, 10 × 10 × 1 and 3 × 3 × 1 *k*-point meshes were used to sample the Brillouin zone for pristine and defective systems, respectively.

## Online content

Any methods, additional references, Nature Portfolio reporting summaries, source data, extended data, supplementary information, acknowledgements, peer review information; details of author contributions and competing interests; and statements of data and code availability are available at 10.1038/s41565-023-01489-x.

### Supplementary information

Supplementary Information Supplementary Notes 1–8, Figs. 1–15 and references.

## Data Availability

The data that support the findings of this study are available on Zenodo at 10.5281/zenodo.8128427.
