# Supplementary material for: Electrical detection of the flat-band dispersion in van der Waals field-effect structures
Source: Nat Nanotechnol. 2023 Aug 17;18(12):1416–22. doi: 10.1038/s41565-023-01489-x (PMC10716041; doi:10.1038/s41565-023-01489-x)
Supplement: Supplementary file 1 — Supplementary Notes 1–8, Figs. 1–15 and references. [file 41565_2023_1489_MOESM1_ESM.pdf]

nature nanotechnology

Article

https://doi.org/10.1038/s41565-023-01489-x

Electrical detection of the flat-band dispersion in van der Waals field-effect structures

In the format provided by the authors and unedited

Table of contents:

1. Carrier effective masses of few-layer InSe and band alignment 2
2. Layer-dependent electrical conductivity of InSe 4
3. Modeling tunneling currents 5
4. Photoluminescence spectroscopy of few-layer InSe..9
5. Bandgap normalization and tunneling photocurrent in 5L-InSe..................................11
6. Tunneling photocurrent in a TMDC heterostructure 12
7. Device fabrication and morphology...14
8. On tunneling currents with flat-band systems 17

References...18

1. Carrier effective masses of few-layer InSe and band alignment

The band alignment of few-layer graphite (FLG)/*N*L-InSe/hBN is obtained from our calculations as described in the Methods section in the main text, with *N* (number of layers) ranging from 1 to 6 (Fig. S1). For all thicknesses, the FLG energy level lies close to the conduction band of *N*L-InSe. Thus, due to the difference in work functions, the Schottky barrier is negligible on the n-side, whereas a sizeable barrier (>0.34eV) is present on the p-side. The band misalignment between hBN and InSe is consistent with the absence of a tunneling current for electrons. On the other hand, the separation between the VBM of InSe and hBN is on the order of hundreds of meVs, allowing a tunneling current with a detectable amplitude according to the mechanisms described in the main text. By employing a hybrid functional in DFT calculations, we obtain bandgap energies that are consistent with the layer-dependent neutral exciton emission energies obtained from PL measurements (Fig. S4).

From the calculated band dispersions of *N*L-InSe, we can compute the effective masses of electrons and holes in InSe, shown in Table S1, yielding values consistent with those reported in the literature.[1–3] The reported masses are obtained as an average between the different directions in the Brillouin zone, from Γ to K, and from Γ to M.

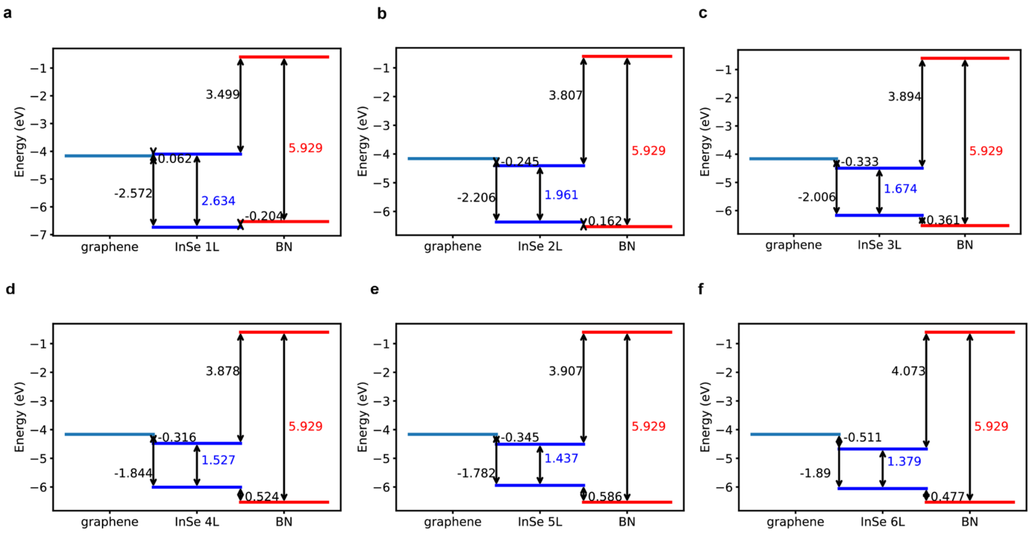

Figure S1. a-f, Band alignment of Graphene/InSe/hBN for different number of InSe layers. InSe slabs with thicknesses ranging from 1L to 6L are considered. The reported values are obtained by DFT simulations as presented in the Methods section of the main text. E = 0 refers to the vacuum level.

Table S1. Effective masses of charge carriers computed for InSe slabs with thicknesses ranging from 1L to 6L.

Number of Layers	Electron effective mass (m_0)	Hole effective mass (m_0)
**1**	0.191	1.726
**2**	0.156	1.076
**3**	0.142	1.003
**4**	0.135	0.951
**5**	0.132	0.929
**6**	0.131	0.913

The shown quantities are the average values of the effective mass calculated for different directions in the Brillouin zone (Γ to K, and Γ to M).

Furthermore, we compute the energy levels of Selenium vacancy (V_{Se}) in 3L InSe. This corresponds to the highest number of layers for which we can successfully reach convergence of the employed computational methods when accounting for defective states. Among the different kinds of defects, we study the effect of V_{Se} because it has the lowest formation energy in InSe[4], thus being the most likely to contribute to the presence of confined states. We find that V_{Se} produces three energy levels located within the bandgap, two of which lie near the CB and one is close to the VB, as shown in Fig. S3 and reported in the main text.

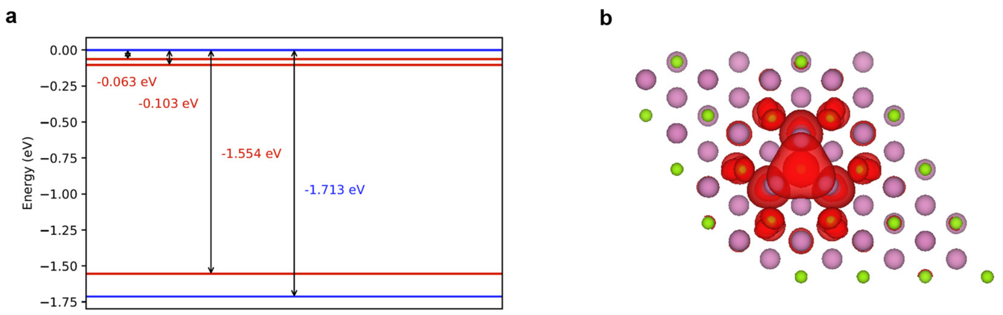

Figure S2. Defect states in 3L-InSe. **a,** Calculated band diagram for 3L InSe with V_{Se}. The energies of defect states due to the selenium vacancy are shown in red, while the energies of band extrema are indicated in blue. The V_{Se} causes 3 states to appear within the bandgap, with two states close to the conduction band and one state close to valence band. **b,** Partial charge density associated with the V_{Se} localized state near the valence band maximum shown in the InSe lattice.

2. Layer-dependent electrical conductivity of InSe

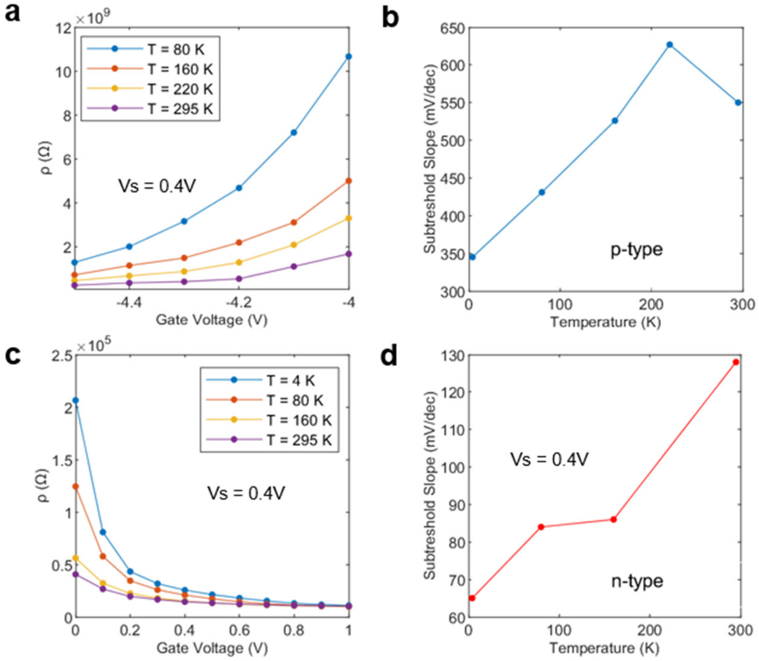

Figure S3. Two-terminal transport in a 6L InSe channel. **a,** P-type resistance as a function of gate voltage for different temperatures for the 6L device (b). **b,** Subthreshold slope (SS) calculated for hole transport as a function of temperature. **c,** N-type two-terminal resistance as a function of V_G at different temperatures. **d,** Subthreshold slope referred to electron transport as a function of T.

We have performed field-effect transport measurements for 6L and 3L InSe devices and summarize the results on Fig. S4. N-type transport is characterized by a decreasing resistance when the semiconductor enters the electron conduction regime, consistently with the literature on InSe[5]. Both n and p-type subthreshold slopes (SS) are shown to increase with temperature (Fig. S3c and Fig. S3e). By comparing the magnitude of the subthreshold resistance variation, we note that resistance of hole transport is significantly higher than the electron counterpart at all temperatures.

As shown in Fig. 4b in the main text, lateral field-effect transport for holes yields a SS lower in 3L with respect to 6L. This can be explained referring to the layer-dependent properties in InSe. The subthreshold slope in a semiconducting layered material is related to its quantum capacitance by:

$$SS_p = log\frac{kT}{q}\left(1+\frac{C_Q}{C_{ox}}\right) \tag{1}$$

In p-type conduction within the VB, we can consider $C_Q \propto m_h$. As shown in Supplementary Table 1, the hole effective mass in NL-InSe decreases with N. Thus, NL-InSe samples with higher hole effective masses are expected to yield higher SS_p values, resulting in $SS_p^{3L} > SS_p^{6L}$, as shown in Fig. S3c.

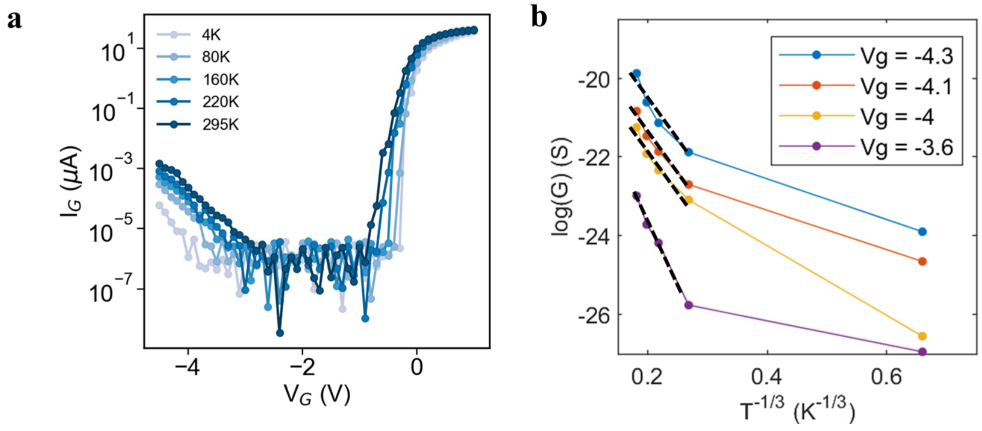

Figure S4. Two-terminal transport in 6L-layer InSe plotted for different temperatures. **a,** Ambipolar transport in 6L InSe shown in logarithmic scale. **b,** P-type conductivity in logarithmic scale as a function of $T^{-1/3}$. A change in trend is visible at around T = 80K, with a linear slope for higher T indicating the 2D Mott variable range hopping regime. The results are shown for different values of V_G.

From the temperature dependence of the transport characteristic in 6L InSe (Fig. S4a), we observe a sizeable shift in the subthreshold onset voltage for both n-type and p-type current. By plotting the logarithm of the conductance *vs*. $T^{-1/3}$, we show a change in trend between temperatures lower and higher than 80K (Fig. S4b). A linear relationship in the T < 80 K temperature region is highlighted for different values of V_G. This behavior is consistent with thermally-activated transport mechanisms based on defective states in the semiconductor, such as the 2D Mott variable range hopping regime, previously observed for other 2D materials[6–8]. As discussed by Qiu *et al*.[6], a temperature-dependent conductance such as the one shown in Fig. S4b cannot be attributed to the thermally activated conduction over the Schottky barrier at the contacts. In fact, the characteristic temperature at which the transport mechanism changes corresponds to an equivalent thermal energy of few meVs (~ 7 meV), which is orders of magnitude lower than the Schottky barrier for holes in few-layer InSe (Supplementary Note 1)[9].

3. Modeling tunneling currents

The tunneling current I can be described considering the tunneling probability across a barrier $T(E, eV, r)$, the density of states of the metal $\rho_M(r, E)$, and that of the second material involved $\rho_S(r, E)$:

$$I \propto \int_0^{eV} \rho_S(r, E)\rho_M(r, E - eV)T(E, eV, r)dE \qquad (2)$$

Within the context of field-effect devices, Simmons' approximation describes tunneling currents through an insulating barrier by delineating two distinct regimes, namely direct tunneling (DT) and Fowler-Nordheim tunneling (FNT)[10]. In metal-insulator-metal (MIM) diodes, the two regimes correspond to trapezoidal and triangular insulating barriers[11]. An increasing bias leads to a shift to the triangular case, inducing an increase in the tunneling

current with respect to the voltage difference between the two electrodes V_G. The two regimes can be extracted from Eq. 2 for MIM devices leading to the following equations:

$$I_{DT} = \frac{A\sqrt{m\phi_B}q^2V_G}{h^2d} exp\left[\frac{-4\pi\sqrt{m^*\phi_B}d}{h}\right] \quad (3)$$

$$I_{FNT} = \frac{Aq^2mV_G^2}{8\pi h\phi_B d^2m^*} exp\left[\frac{-8\pi\sqrt{2m^*}\phi_B^{3/2}d}{3hqV_G}\right] \quad (4)$$

where A is the gated active area, q and m^* are the electron charge and mass, d is the insulating barrier width and m^* is the effective mass of the tunneling medium[12]. In MIM devices, the transition voltage V_{TRAN} is defined as the voltage difference between the metal layers necessary to reach a triangular barrier profile and transition from DT to FNT. Thus, V_{TRAN} is equivalent to the insulator barrier height ($V_{TRAN}^{MIM} = \phi_B/e$). Within this context, V_{TRAN} can be deduced experimentally from the inflection point observed in $ln(I_{FNT}/V_G^2)$ as a function of $1/V_G$, giving a value on the order of 0.5V in silicon nanosheets[11].

In the case of InSe-based devices, the difference between the InSe and the hBN valence bands is ~ 0.5V (Fig. S1), one order of magnitude lower than the observed transition voltage (V_{TRAN}^{5L} V$\approx 5.2V$). Thus, we exclude the possibility that our observed signal could be due to a change in the energy barrier shape. Nonetheless, V_{TRAN} can still be used as a signature position of the change in tunneling regimes.

The tunneling probability under the WKB approximation for a triangular barrier takes the general form of:

$$T \propto e^{\left(\frac{-2d\sqrt{2m^*}}{\hbar}\sqrt{\frac{\Phi s+\Phi_M}{2}+\frac{eV}{2}-E}\right)} \quad (5)$$

where m^* is the effective mass of the tunneling medium (hBN in our case), d is the thickness of the tunneling medium, Φs is the sample work function, and Φ_M is the metal work function. The tunneling relationship for photoelectric emission can be generalized for any tunneling process involving a triangular barrier by the following[12–14]:

$$I_{FNT} \propto V_G^2 exp\left[\frac{-8\pi\sqrt{2m^*}\phi_B^{3/2}d}{3hqV_G}\right] \quad (6)$$

Due to its general validity, Eq. 6 has been widely employed when modeling tunneling photocurrents between semiconductors and metallic gates[13–16]. Here, the effective tunneling barrier ϕ_B represents the insulating barrier cumulatively felt by carriers when tunneling. ϕ_B can be extracted by a linear fit of $ln(I_{FNT}/V_G^2)$ as a function of $1/V_G$. However, in photo-assisted tunneling processes involving 2D materials, both V_{TRAN} and ϕ_B depend on the related physical processes and need to be specifically addressed based on the dominant exciton dynamics and on the semiconductor DOS. In our case, for the *d* parameter we used 20 nm, 25 nm, 18 nm as the hBN thicknesses of the 3L, 5L, and 6L devices, respectively. In the main text, we elucidate on the interpretation of the tunneling photocurrent with few-layer InSe as an active

medium. Moreover, in Supplementary Note 6, we report on photo-assisted processes in a TMDC heterostructure for comparison.

In STM measurements[17,18], the derivative of the tunneling current with respect to the tip bias is used as a means of extracting information on the electrical properties of the sample:

$$\frac{dI_G}{dV_G} \propto \rho_S(r,eV)\rho_M(r,0)T(eV,eV,r) + \int_0^{eV} \rho_S(r,E)\rho_M(r,E-eV)\frac{dT(E,eV,r)}{dV}dE \quad (7)$$

This equation is independent of tunneling mechanisms and can be applied to our devices by considering InSe and few-layer graphite for ρ_S and ρ_M, respectively. In Fig. S5a, we show the dI_G/dV_G signal as a function of V_G for the 5L and 6L samples, featuring a peak rising in proximity of the corresponding V_{TRAN} values.

From Eq. 5, we note that T is influenced by the change in barrier shape, monotonically dependent on the sample bias, and independent from ρ_S. From Eq. 4, it follows that extracting ρ_S would require knowing both ρ_M and T. The ρ_M is commonly considered as constant and T as slowly varying with respect to the sample bias, as long as no change in barrier shape (trapezoidal to triangular) is encountered in the region of interest[19]. As delineated by Feenstra *et al.*[20], we can investigate the tunneling behavior independently of T by taking the ratio between the differential conductance and the tunneling conductance itself:

$$\frac{\frac{dI_G}{dV_G}}{\frac{I_G}{V_G}} = \frac{\rho_S(r,eV)\rho_M(r,0) + \int_0^{eV} \frac{\rho_S(r,E)\rho_M(r,E-eV)}{T(eV,eV,r)}\frac{dT(E,eV,r)}{dV}dE}{\frac{1}{eV}\int_0^{eV} \rho_S(r,E)\rho_M(r,E-eV)\frac{T(E,eV,r)}{T(eV,eV,r)}dE} \quad (8)$$

As previously noted, the transmission probability in our sample changes when the barrier becomes triangular at $V_G = (\Phi_{InSe} - \Phi_{hBN})/e \simeq -0.5V$. Although such transition occurs, no onset is observed in the tunneling current at the corresponding V_G, indicating that the change in T is not driving the measured signal increase. On the other hand, we observe an exponential onset in I when the Fermi level reaches the flat band located at the VBM, as confirmed by lateral field-effect transport and PL measurements (see main text). Our results are represented in Fig. S5 following Eq. 7 and Eq. 8.

The presence of a peak in the $\frac{dI_G}{dV_G} / \frac{I_G}{V_G}$ signal further confirms that the measured exponential rise in the tunneling currents in proximity of V_{TRAN} is a consequence of a sharp increase in the material DOS. Differently from traditional local STS techniques[21], our measurements involve the entire InSe flake region, since we are considering an average over the gated area. We note that this is possible with few-layer InSe in particular, thanks to the presence of a largely asymmetric band alignment with respect to hBN (Supplementary Note 1), allowing us to probe changes at the VBM specifically. On the other hand, tunneling spectroscopy measurements carried out in twisted systems investigate flat bands with respect to the scanning tip position along the moiré patterns of the heterostack[22–27].

To further elucidate the behavior of the tunneling current measured in dark conditions, we have performed temperature-dependent measurements, starting from 4 K up to room temperature. The results presented in Fig. S6 show a clear trend of the tunneling current, where the magnitude of the current increases at increasing temperatures. We note that, at 4 K it is already possible to distinguish the change in trend at the flat band position, which was not present at 100 mK as shown in Fig. 4 of the main text. We ascribe this difference to the broadening of the Fermi-Dirac distribution, which is much narrower at 100 mK, and sufficiently broad at 4 K to allow for the observation of the effect.

Due to its properties and based on the previous reasoning, InSe holds promise for reproducible flat-band systems that allow for the investigation of correlated physics by optoelectronic measurements in field-effect structures.

We note that laser illumination could hinder the observation of emerging phenomena in flat-band systems due to the rise of the electron/hole gas temperature. Thus, the possibility of employing low laser power or fully dark environments to detect the singularity position would be beneficial for future work on flat-band systems. Our dark measurements demonstrate that laser illumination is not strictly needed for the detection of the van Hove singularity position. Therefore, by employing thinner dielectric barriers, exponentially higher signals can be obtained at extremely low temperatures, motivating the use of tunneling currents for the exploration of flat-band systems and their emergent phenomena down to $T < 100$ mK.

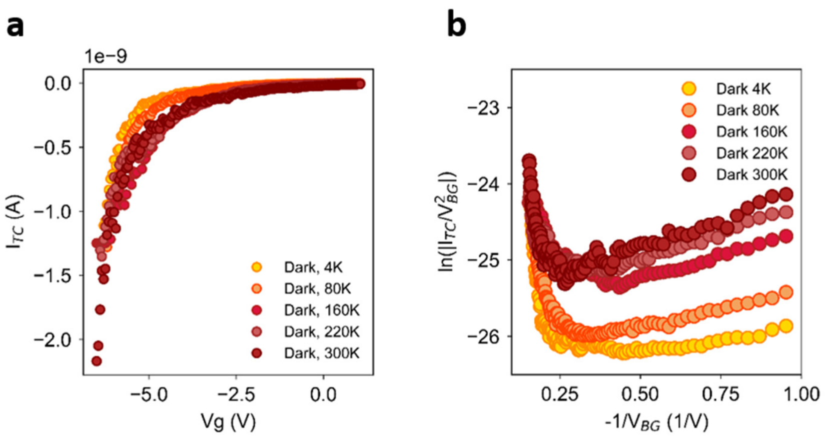

Figure S6. Tunneling current signal in the 3L device at different temperatures without laser illumination. **a,** Temperature-dependent dark tunneling current from 4.5 K to 300 K, plotted in linear scale as a function of V_G. **b,** Temperature-dependent dark tunneling current from 4.5 K to 300 K, plotted in the Simmons scale as a function of V_G.

4. Photoluminescence spectroscopy of few-layer InSe

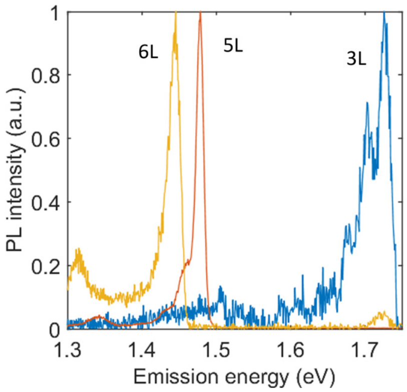

Figure S7. Layer-dependent photoluminescence (PL) in InSe. The neutral exciton peak is reported for 3L, 5L and 6L samples. The layer-dependence of the emitted energy peak for X_0 is consistent with the calculated bandgaps (Supplementary Note 1).

Photoluminescence spectroscopy was carried out for all the devices described in the main text. Gate-dependent data was obtained for the 5L and 6L samples, shown in Fig. S8 and Fig. 3a respectively. The weakness of the PL emission in the 3L sample did not allow us to extract a gate-dependent behavior. However, we have been able to record the PL emission of X_0. In Fig. S7, we show the X_0 PL emission for the 3L, 5L and 6L devices.

As previously reported in the literature, few-layer InSe is characterized by a PL emission energy that increases with a decreasing number of layers[5,28]. Moreover, optical absorption in few-layer InSe with normally incident laser light is not influenced by the A-exciton resonance[29], and we have not observed gate-dependency of absorbance. If we consider the neutral exciton emission X_0, we observe an emission energy of approximately 1.75 eV, 1.48 eV and 1.43 eV for the 3L, 5L and 6L devices, respectively. By comparing the PL emission of X_0 with the reported literature, we've been able to identify the number of layers of the InSe samples shown in this work.

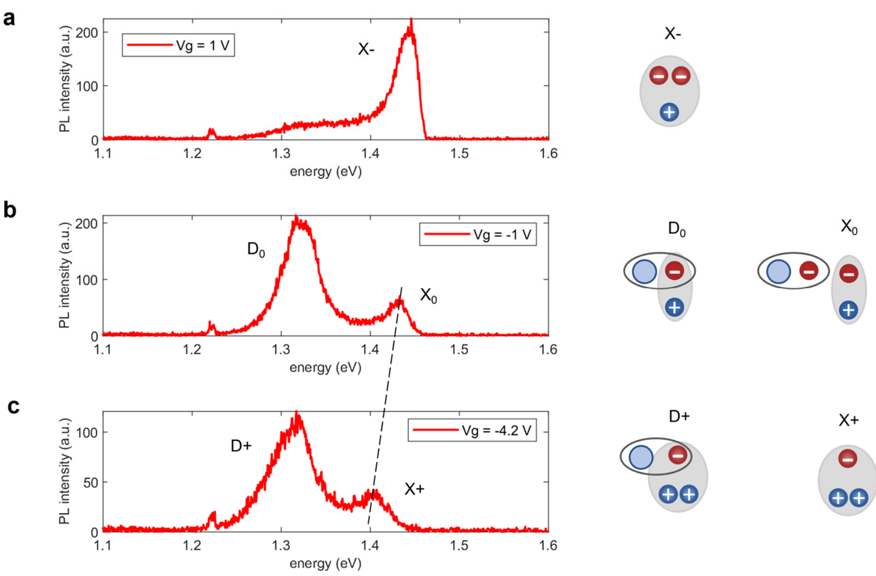

Figure S8. Photoluminescence spectroscopy line-cuts at different gate voltage values**. a,** PL emission of the negative trion peak measured with $V_G = 1\,\mathrm{V}$. **b,** PL intensity of neutral exciton (X_0) and defect bound exciton obtained within the bandgap at $V_G = -1\,\mathrm{V}$. **c,** Defect bound charged exciton peak (D_+), and positive trion (X_+) at $V_G = -4.2\,\mathrm{V}$. The black dashed line highlights the sizeable redshift in emission energy of the highest excitonic peak, which is related to the bandgap renormalization process at the valence band edge.

Figure S8 shows three linecuts of gate-dependent PL data (Fig. 3a in the main text) for different values of V_G. The chosen gate voltages correspond to the main charge configuration regions of our interest in this work, namely n-type, in-gap, and p-type. In the n-type region (Fig. S8a), the negative trion emits at 1.42 eV, with a PL signal tail appearing at lower energies due to defect bound trion states, as discussed in our previous work[30]. Within the bandgap, at $V_G = -1$ V (Fig. S8b), the neutral exciton peak (X_0) emits at 1.43 eV, and the defect bound exciton peak (D_0) shows at around 1.32 eV, roughly 100 meV below the neutral exciton. In the p-type region, the defect bound excitons gradually disappears, together with a defect bound trion tail (D_+), while the positive trion (X_+) features a sizeable redshift due to bandgap renormalization and a reduction in maximum PL signal.

5. Bandgap normalization and tunneling photocurrent in 5L-InSe

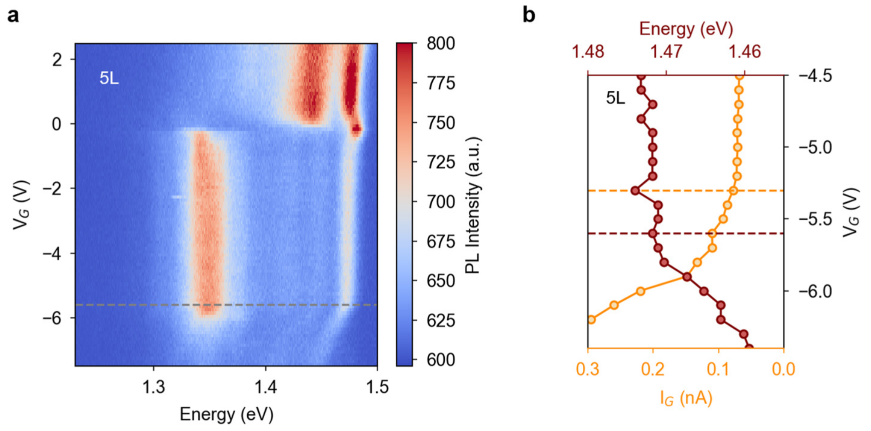

Figure S9. a, Gate-dependent PL emission in the 5L device. The behavior of the obtained spectra is comparable to that observed in the 6L sample (Fig. 3a in the main text). The start of the bandgap renormalization process within the valence band is indicated by a dashed grey line. **b,** Gate-dependence of the highest exciton peak energy (red) and the tunneling photocurrent (yellow). V_{BGR}^{5L} and V_{ON}^{5L}, as defined in the main text, are indicated by red and yellow dashed lines, respectively.

In Fig. S9a, we report the gate-dependent PL emission of a 5L-InSe device at 4K. The measurements were taken as described in the Methods section of the main text. We note that the excitonic behavior for 5L is compatible with that shown in Fig. 3a (6L device). In the 5L sample, for $V_G < -5.6$ V a linear shift of the highest-energy exciton peak is observed, according to the bandgap renormalization mechanism occurring when entering the van Hove singularity at the valence band. In Fig. S9b, we compare the free-exciton PL emission to the tunneling photocurrent with respect to gate voltage when reaching the VBM. As described in Supplementary Note 4, the highest-energy exciton peak is defined as X_0 for $-5.6\text{ V} < V_G < -0.7\text{ V}$ and X+ for $V_G < -5.6$ V. We identify $V_{BGR}^{5L} = -5.6$ V and $V_{TRAN}^{5L} = -5.3$ V, respectively, as described in the main text and reported in Fig. 3e. We note that the tunneling photocurrent onset is better extracted from the transition point in the FNT scale (Fig. 2b).

6. Tunneling photocurrent in a TMDC heterostructure

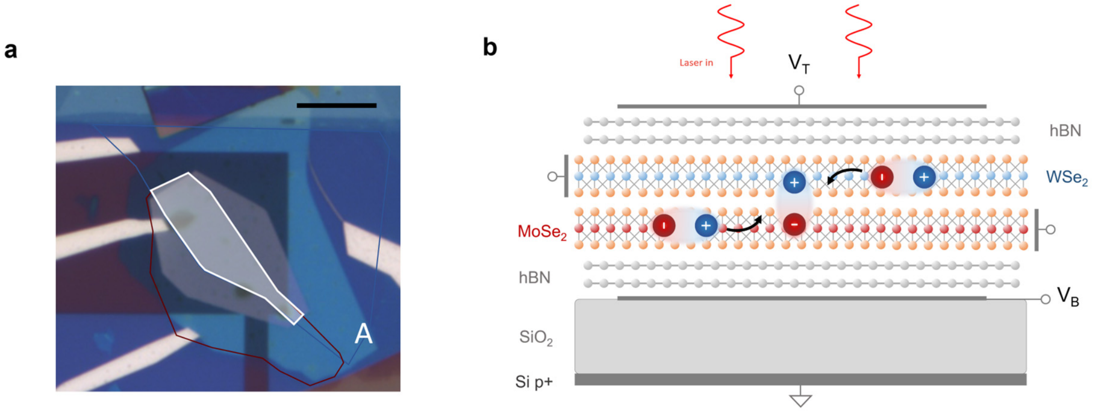

Figure S10. a-b, False-colour optical image (a) and device schematic (b) of an hBN-encapsulated $WSe_2/MoSe_2$ heterobilayer. The WSe_2 and $MoSe_2$ monolayers are highlighted in blue and red, respectively, while the gated heterostructure area is shown in white. Optically-excited type-II heterobilayers host both spatially direct and indirect (interlayer) excitonic species[31–33].

Anomalous Fowler-Nordheim tunneling through thick hBN layers has been reported in other van der Waals structures, with effective tunneling barrier height values that are much lower (~100 meV) than the predicted band alignment between TMDC and hBN layers (1 – 2 eV)[13–16]. Various mechanisms have been indicated as responsible for phototunneling currents through hBN, with the formation of hot carriers assisted by exciton-exciton annihilation or Auger processes as the main candidates.

We have fabricated van der Waals heterostructures with TMDCs deterministically stacked by mechanical wet transfer techniques (device A). The optically active medium consists of an hBN-encapsulated $MoSe_2/WSe_2$ heterobilayer (Fig. S10). Contacts and gates have been fabricated as described in the Methods section in the main text. We have investigated tunneling currents through the thick top (20 nm) and bottom (25 nm) hBN layers in the sample shown on Fig. S10a. First, we have performed measurements in dark by sweeping the bottom-gate voltage and recording the tunneling current as a function of V_G. At sufficiently high gate voltages, the tunneling mechanism changes from direct (DT) to Fowler-Nordheim (FNT), allowing us to estimate the tunneling barrier height by fitting to FNT in triangular potentials (Supplementary Note 3)[13]. In this case, we consider a current of electrons tunneling from the bottom gate to the TMDCs (Fig. 9a). We have extracted a conduction-band offset of approximately 2.23 eV between the bottom gate and the hBN barrier.

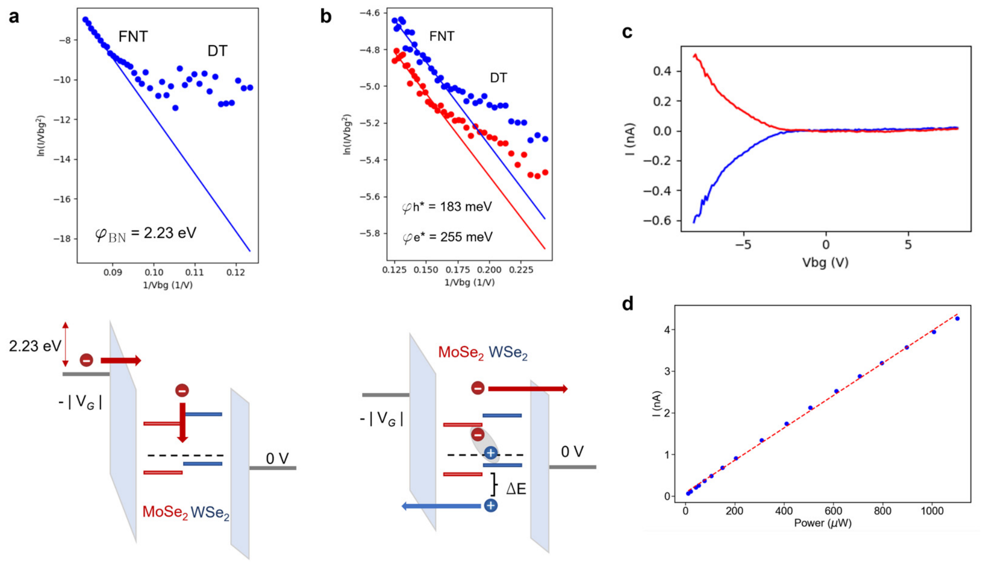

Figure S11. Tunneling photocurrent in a TMDC heterostructure. **a,** Fowler-Nordheim tunneling (FNT) and direct tunneling (DT) of electrons from the bottom gate to the TMDCs. The plot is based on a commonly used data representation for FNT fitting[13]. The extracted barrier height between the bottom gate and the hBN is estimated as 2.23eV (top). A schematic representation indicates the voltage configuration and band bending in presence of an applied bottom gate voltage, with the top gate kept grounded. **b,** Exciton-recombination-assisted tunneling of carriers from the TMDCs to the bottom and top gate electrodes. The low extracted barrier heights indicates that hot carriers are tunneling through the hBN barriers. **c,** Photocurrent as a function V_{BG}. **d,** Linear relationship between the measured photocurrent and the input laser power.

When exposed to laser light, bottom-gate sweeps have been performed as described in the main text for InSe devices. By shining a red laser (647 nm) at sufficiently high power (>10 μW) on the sample, we see FNT at lower V_{BG} than in dark, meaning that a different tunneling mechanism is responsible. Based on the sign of the obtained currents on the bottom and top gates, we attribute the observed phenomenon to holes and electrons, respectively flowing from the TMDCs to the electrodes across the hBN barriers (Fig. S11b). In order to explain such a carrier flow, we must consider non-radiative transfer of exciton energy to resident carriers, that become sufficiently excited to be able to tunnel through a triangular hBN barrier[14–16]. Through FNT fitting, we extract tunnel barrier heights for electrons and holes on the order of 200 meV, which is consistent with a picture involving hot carriers excited in the TMDCs.

Previous reports have distinguished between exciton-exciton annihilation (EEA), trap-assisted and Auger recombination as the main mechanisms responsible for carrier excitation based on the dependence of the tunneling currents with respect to the input laser power[14–16]. Due to the difference between two-exciton and single-exciton mechanisms, quadratic and linear trends have been linked to EEA and Auger processes, respectively. Based on the power-dependent data in Fig. S11d, we have attributed our photocurrent to the latter mechanism.

We note that a smooth change is present between the DT and FNT regimes, consistently with previous reports on tunneling photocurrents with monolayer or multi-layer TMDC structures[14–16]. With a TMDC heterostructure (Fig. S10b), the shift from DT to FNT is related to the progressive bending of the hBN bands with increasing electric fields, with a smooth transition. Instead, with *N*L-InSe, the sharp transition in hole phototunneling is directly related to the steep DOS increase at the van Hove singularity at the valence band edge. This is also

responsible for the change in tunneling mechanisms and the reported bandgap renormalization process, as described in the main text.

7. Device fabrication and morphology

In this section, we present the material, the fabrication and the morphological characterization of the devices considered in this work. First, we exfoliate all the building blocks on SiO_2, apart from the InSe flake, which is exfoliated on PDMS. The optical micrographs of the as-exfoliated flakes is presented in Figure S12.

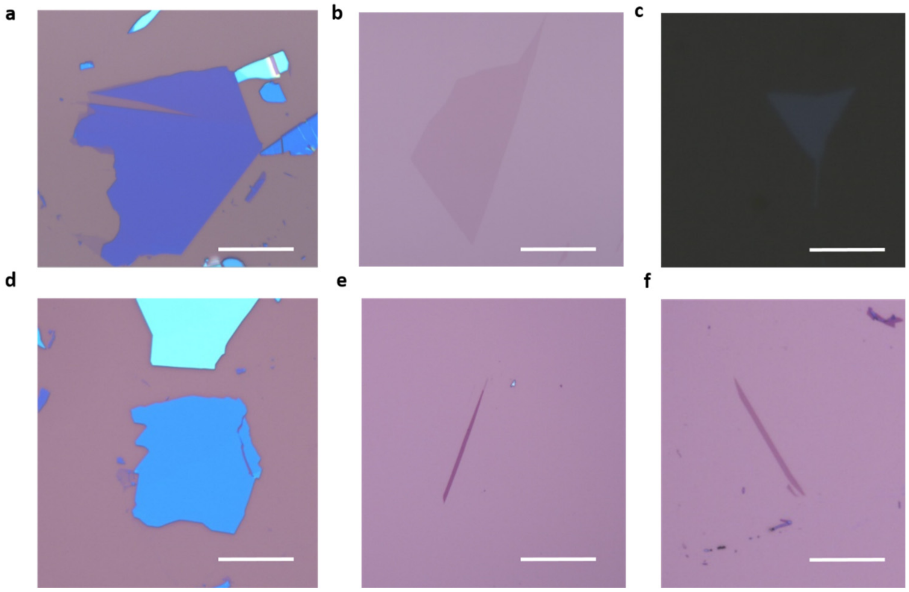

Figure S12. As exfoliated building blocks for the 3L device. **a,** Bottom hBN flake, thickness of 20 nm. **b,** Bottom graphene gate. **c,** Optical image of 3L InSe, thickness 2.5 nm. **d,** Top hBN used to pick up the electrodes and the rest of the structure. **e-f,** Few-layer graphene electrodes. The scale bars are 20 μm.

Once the flakes have been carefully selected under the optical microscope, we perform the assembly by a traditional dry-stacking method. The resulting device is shown in Figure S13a. We perform the same device fabrication for all the devices discussed in this work. Furthermore, the optical images and corresponding hBN thicknesses of the bottom hBN layers for 3L, 5L and 6L devices are all reported in Figure S13.

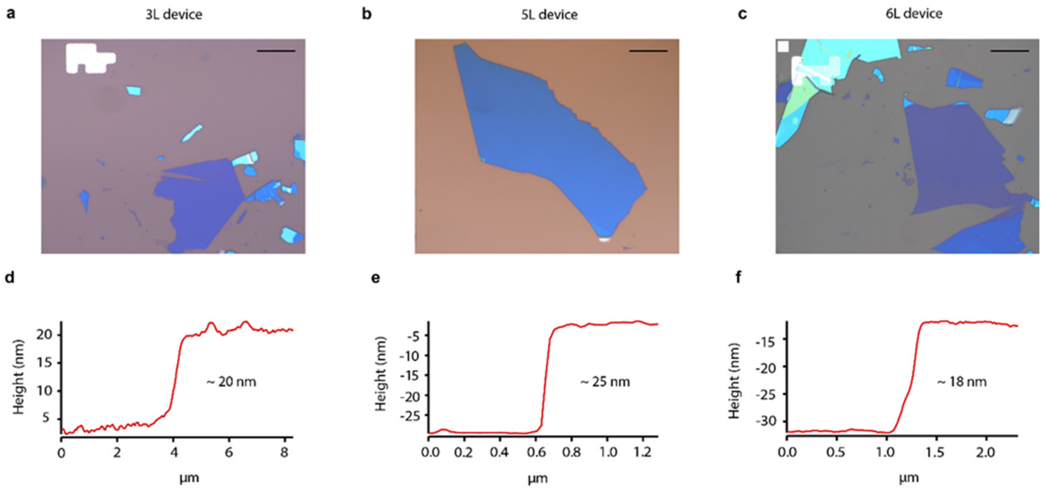

Figure S13. Optical micrographs (a-c) and corresponding AFM thickness measurements (d-f) of the bottom hBN layers for the 3L, 5L and 6L devices, respectively. The scale bars are 25 µm.

To better highlight the reliability of our tunneling current analysis, we show the characterization of our devices by comparing the optical micrograph with the AFM scan, the photoluminescence map, and the tunneling photocurrent map for the 3L and 5L devices in Figures S14 and S15, respectively. These results show that the tunneling current signal is highly uniform across the active areas of the devices.

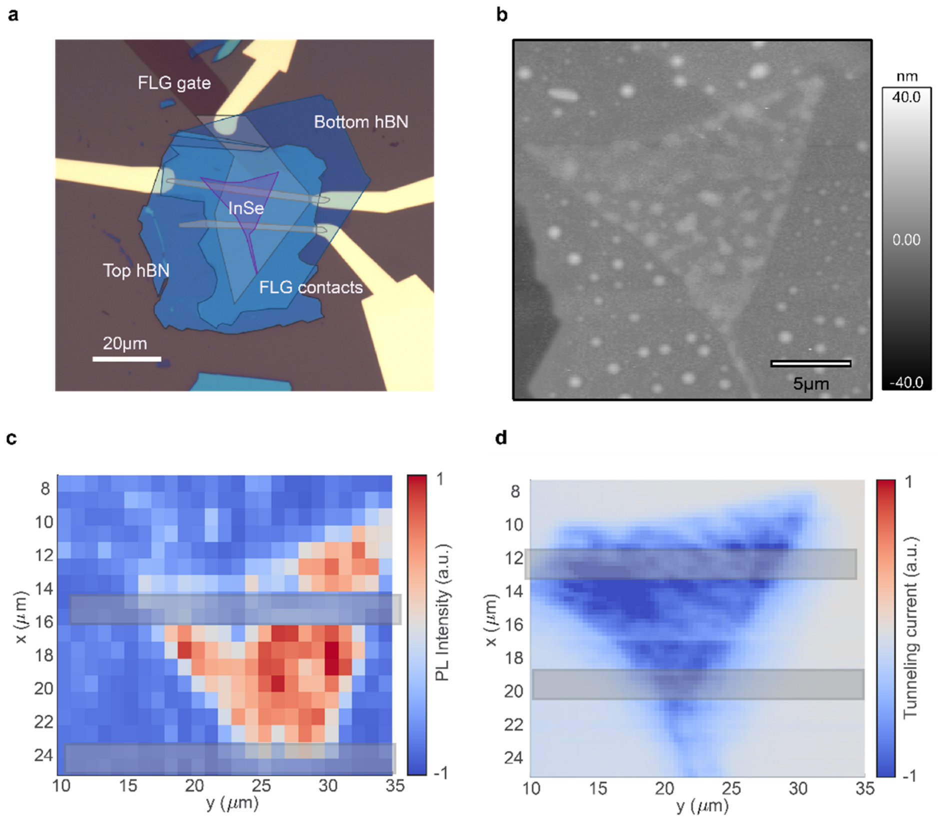

Figure S14. Morphology and characterization of the 3L InSe device. **a,** Optical micrograph of the heterostructure. The flakes are highlighted in false color to better distinguish the building blocks. **b,** AFM morphology scan of the same device. **c,** Photoluminescence map recorded at 4K under 532 nm laser excitation. The lower counts at the center of the device correspond to the graphene electrode, as indicated by the optical micrograph and the grey overlays. **d,** Tunneling photocurrent map recorded with high spatial resolution. The data clearly shows a uniform distribution of the tunneling current across the active area of the device.

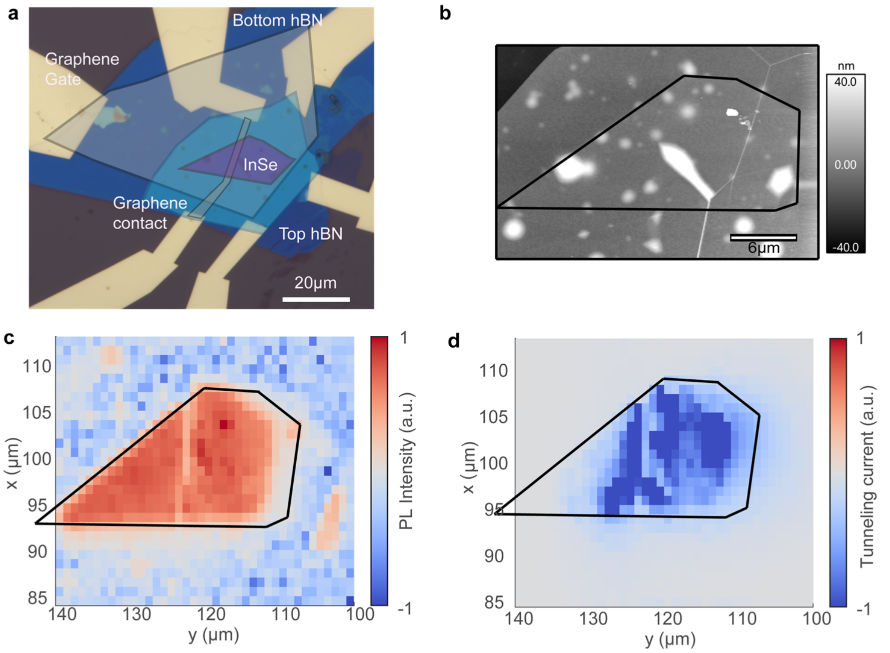

Figure S15. Morphology and characterization of the 5L InSe device. **a,** Optical micrograph of the heterostructure. The flakes are highlighted in false color to better distinguish the building blocks. **b,** AFM morphology scan of the same device. The black contour indicates the 5L InSe. **c,** Photoluminescence map measured under 648 nm laser excitation at 4K. The lower counts at the center of the device correspond to the graphene electrode, as indicated by the optical micrograph. **d,** Tunneling photocurrent map recorded simultaneously with respect to the PL map. The data shows a rather uniform distribution of the tunneling current across the active area of the device.

8. On tunneling currents with flat-band systems

Our method does not strictly require the use of InSe, since it is based on a tunnelling mechanism dictated by the presence of a van Hove singularity with a high density of states. Thus, we can directly determine the generalizability of our method based on the findings in our present work as follows.

The exponential tunnelling mechanism rising at the van Hove singularity position is described by the Equation 6 for both dark and illuminated cases, as detailed in Supplementary Note 3. The increase of V_G is limited by dielectric breakdown. Thus, the critical parameters to obtain a sizeable tunnelling signal independently on V_G are:

- the band alignment between the van Hove singularity energy position and the dielectric band edge (ϕ_B);
- the thickness of the insulating barrier (d);
- the out-of-plane effective mass of the carriers of interest in the dielectric (m^*).

Therefore, our method can be successfully applied to any flat-band system provided that proper device engineering is employed. In particular, the choice of the dielectric and its band alignment with the flat-band system of interest must be designed in order to have measurable signals across the chosen insulating barrier.

Regarding our structures, values of ϕ_B on the order of hundreds of meV have revealed sizeable p-type tunnelling currents for InSe on hBN. In particular, we have shown that a barrier of ~ 400 meV with an hBN thickness of ~ 25 nm is sufficient to obtain sizeable tunnelling hole currents in our structures. Instead, no electron tunneling signals have been measured due to the presence of an effective barrier > 3 eV for electrons. Since we observed hole tunneling currents on the order of tens of nano amperes for InSe with hBN thicknesses that reach ~ 25 nm, comparable or higher signals can be obtained for other flat band systems by using thinner hBN dielectrics in the case of similar band alignments. Further device engineering can be employed to optimize the tunnelling mechanism independently of the dielectric, such as by increasing the active area or the laser excitation power (Figure 3c).

In Supplementary Note 6, we show sizeable tunnelling currents of excited states from both electrons and holes in a $MoSe_2$/WSe_2 heterostructure with a symmetrical band alignment with hBN. However, in this case, the absence of a van Hove singularity does not allow us to observe a drastic change in tunnelling mechanisms. Nonetheless, this study proves that band-alignment engineering allows sizeable currents based on the system of interest and on the dielectric of choice, enabling us to infer the generalization of our method to other materials beyond InSe. Moreover, any dielectric can be chosen to optimize the m^* and ϕ_B factors. In our case, the choice of hBN is motivated by requiring a high-quality encapsulation environment for both lateral transport and optical characterization. Instead, regarding a generalized tunnelling current method for flat-band systems, other dielectrics compliant with the the previously detailed requirements would be eligible. As an example, the widely used high-k dielectric HfO_2 is characterized by hole ($\sim 0.5\, m_0$) and electron ($\sim 0.1\, m_0$) effective masses that are comparable or lower than those of hBN[34,35]. Therefore, HfO_2 could be used as a medium for tunnelling currents with flat band systems if its band alignment with the 2D materials of interest satisfies the requirements detailed above.

References

1. Zólyomi, V., Drummond, N. D. & Fal'ko, V. I. Electrons and phonons in single layers of hexagonal indium chalcogenides from ab initio calculations. *Phys. Rev. B* **89**, 205416 (2014).
2. Mudd, G. W. *et al.* The direct-to-indirect band gap crossover in two-dimensional van der Waals Indium Selenide crystals. *Sci. Rep.* **6**, 39619 (2016).
3. Rybkovskiy, D. V., Osadchy, A. V. & Obraztsova, E. D. Transition from parabolic to ring-shaped valence band maximum in few-layer GaS, GaSe, and InSe. *Phys. Rev. B* **90**, 235302 (2014).
4. Xiao, K. J., Carvalho, A. & Castro Neto, A. H. Defects and oxidation resilience in InSe. *Phys. Rev. B* **96**, 054112 (2017).
5. Bandurin, D. A. *et al.* High electron mobility, quantum Hall effect and anomalous optical response in atomically thin InSe. *Nat. Nanotechnol.* **12**, 223–227 (2017).
6. Qiu, H. *et al.* Hopping transport through defect-induced localized states in molybdenum disulphide. *Nat. Commun.* **4**, 2642 (2013).
7. Xue, J., Huang, S., Wang, J.-Y. & Xu, H. Q. Mott variable-range hopping transport in a MoS_2 nanoflake. *RSC Adv.* **9**, 17885–17890 (2019).
8. Lösche, A. N. F. MOTT, E. A. DAVIS. Electronic Processes in Non-Crystalline Materials Clarendon-Press, Oxford 1971 437 Seiten. £ 7,50. *Krist. Tech.* **7**, K55–K56 (1972).
9. Mudd, G. W. *et al.* High Broad-Band Photoresponsivity of Mechanically Formed InSe–Graphene van der Waals Heterostructures. *Adv. Mater.* **27**, 3760–3766 (2015).
10. Simmons, J. G. Generalized Formula for the Electric Tunnel Effect between Similar Electrodes Separated by a Thin Insulating Film. *J. Appl. Phys.* **34**, 1793–1803 (1963).

11. Ikuno, T. *et al.* Electron transport properties of Si nanosheets: Transition from direct tunneling to Fowler-Nordheim tunneling. *Appl. Phys. Lett.* **99**, 023107 (2011).
12. Ma, Q. *et al.* Tuning ultrafast electron thermalization pathways in a van der Waals heterostructure. *Nat. Phys.* **12**, 455–459 (2016).
13. Vu, Q. A. *et al.* Tuning Carrier Tunneling in van der Waals Heterostructures for Ultrahigh Detectivity. *Nano Lett.* **17**, 453–459 (2017).
14. Chow, C. M. E. *et al.* Monolayer Semiconductor Auger Detector. *Nano Lett.* **20**, 5538–5543 (2020).
15. Linardy, E. *et al.* Harnessing Exciton–Exciton Annihilation in Two-Dimensional Semiconductors. *Nano Lett.* **20**, 1647–1653 (2020).
16. Sushko, A. *et al.* Asymmetric photoelectric effect: Auger-assisted hot hole photocurrents in transition metal dichalcogenides. *Nanophotonics* **10**, 105–113 (2021).
17. Chen, C. J. *Introduction to Scanning Tunneling Microscopy*. (Oxford University Press, 2016).
18. Bonnell, D. Scanning Probe Microscopy and Spectroscopy: Theory, Techniques, and Applications. (John Wiley & Sons, 2000).
19. Books: Scanning Probe Microscopy and Spectroscopy: Methods and Applications by Roland Wiesendanger, R. Wiesendanger (9780521428477), Best Reviews 2022.
20. Feenstra, R. M., Stroscio, J. A. & Fein, A. P. Tunneling spectroscopy of the Si(111)2 × 1 surface. *Surf. Sci.* **181**, 295–306 (1987).
21. Fu, W. *et al.* An Anomalous Magneto-Optic Effect in Epitaxial Indium Selenide Layers. *Nano Lett.* **20**, 5330–5338 (2020).
22. Choi, Y. *et al.* Electronic correlations in twisted bilayer graphene near the magic angle. *Nat. Phys.* **15**, 1174–1180 (2019).
23. Andrei, E. Y. & MacDonald, A. H. Graphene bilayers with a twist. *Nat. Mater.* **19**, 1265–1275 (2020).
24. Xie, Y. *et al.* Spectroscopic signatures of many-body correlations in magic-angle twisted bilayer graphene. *Nature* **572**, 101–105 (2019).
25. Li, H. *et al.* Imaging moiré flat bands in three-dimensional reconstructed WSe_2/WS_2 superlattices. *Nat. Mater.* **20**, 945–950 (2021).
26. Jiang, Y. *et al.* Charge order and broken rotational symmetry in magic-angle twisted bilayer graphene. *Nature* **573**, 91–95 (2019).
27. Kerelsky, A. *et al.* Maximized electron interactions at the magic angle in twisted bilayer graphene. *Nature* **572**, 95–100 (2019).
28. Song, C. *et al.* Largely Tunable Band Structures of Few-Layer InSe by Uniaxial Strain. *ACS Appl. Mater. Interfaces* **10**, 3994–4000 (2018).
29. Magorrian, S. J., Zólyomi, V. & Fal'ko, V. I. Electronic and optical properties of two-dimensional InSe from a DFT-parametrized tight-binding model. *Phys. Rev. B* **94**, 245431 (2016).
30. Pasquale, G. *et al.* Flat-band-induced many-body interactions and exciton complexes in a layered semiconductor. Preprint at https://doi.org/10.48550/arXiv.2207.13472 (2022).
31. Unuchek, D. *et al.* Room-temperature electrical control of exciton flux in a van der Waals heterostructure. *Nature* **560**, 340–344 (2018).
32. Ciarrocchi, A. *et al.* Polarization switching and electrical control of interlayer excitons in two-dimensional van der Waals heterostructures. *Nat. Photonics* **13**, 131 (2019).
33. Unuchek, D. *et al.* Valley-polarized exciton currents in a van der Waals heterostructure. *Nat. Nanotechnol.* **14**, 1104–1109 (2019).
34. Garcia, J. C. *et al.* Effective masses and complex dielectric function of cubic HfO_2. *Appl. Phys. Lett.* **85**, 5022–5024 (2004).
35. Monaghan, S., Hurley, P. K., Cherkaoui, K., Negara, M. A. & Schenk, A. Determination of electron effective mass and electron affinity in HfO_2 using MOS and MOSFET structures. *Solid-State Electron.* **53**, 438–444 (2009).
